# Core functional traits of bacterial communities in the Upper Mississippi River show limited variation in response to land cover

**DOI:** 10.3389/fmicb.2014.00414

**Published:** 2014-08-08

**Authors:** Christopher Staley, Trevor J. Gould, Ping Wang, Jane Phillips, James B. Cotner, Michael J. Sadowsky

**Affiliations:** ^1^BioTechnology Institute, University of MinnesotaSt. Paul, MN, USA; ^2^Biology Program, University of MinnesotaSt. Paul, MN, USA; ^3^Department of Ecology, Evolution and Behavior, University of MinnesotaSt. Paul, MN, USA; ^4^Department of Soil, Water and Climate, University of MinnesotaSt. Paul, MN, USA

**Keywords:** functional diversity, metagenomics, microbial community, microbial biogeography, Mississippi River, recreational water

## Abstract

Taxonomic characterization of environmental microbial communities via high-throughput DNA sequencing has revealed that patterns in microbial biogeography affect community structure. However, shifts in functional diversity related to variation in taxonomic composition are poorly understood. To overcome limitations due to the prohibitive cost of high-depth metagenomic sequencing, tools to infer functional diversity based on phylogenetic distributions of functional traits have been developed. In this study we characterized functional microbial diversity at 11 sites along the Mississippi River in Minnesota using both metagenomic sequencing and functional-inference-based (PICRUSt) approaches. This allowed us to determine how distance and variation in land cover throughout the river influenced the distribution of functional traits, as well as to validate PICRUSt inferences. The distribution and abundance of functional traits, by metagenomic analysis, were similar among sites, with a median standard deviation of 0.0002% among tier 3 functions in KEGG. Overall inferred functional variation was significantly different (*P* ≤ 0.035) between two water basins surrounded by agricultural vs. developed land cover, and abundances of bacterial orders that correlated with functional traits by metagenomic analysis were greater where abundances of the trait were inferred to be higher. PICRUSt inferences were significantly correlated (*r* = 0.147, *P* = 1.80 × 10^−30^) with metagenomic annotations. Discrepancies between metagenomic and PICRUSt taxonomic-functional relationships, however, suggested potential functional redundancy among abundant and rare taxa that impeded the ability to accurately assess unique functional traits among rare taxa at this sequencing depth. Results of this study suggest that a suite of “core functional traits” is conserved throughout the river and distributions of functional traits, rather than specific taxa, may shift in response to environmental heterogeneity.

## Introduction

Over the last 30 years, sequencing of the 16S rRNA gene has allowed for expansive characterization of microbial biodiversity from a variety of hosts and habitats (Olsen et al., 1986). Early reviews of available 16S rDNA datasets, generated primarily from studies employing random clone libraries, revealed that several major bacterial phyla, including the α-, β-, and *γ*-*Proteobacteria, Actinobacteria, Bacteroidetes*, and *Cyanobacteria*, comprised ubiquitous freshwater lineages (Gløckner et al., 2000; Zwart et al., 2002). More recently, the emergence of next-generation sequencing technologies has also supported the predominance of these groups in freshwater riverine ecosystems, and has allowed for assessments of variability among bacterial communities (Ghai et al., 2011; Portillo et al., 2012; Staley et al., 2013). These studies have demonstrated that the taxonomic bacterial community structure in aquatic environments is, at least in part, shaped by gradients of physicochemical and biotic parameters (Gilbert et al., 2009; Fortunato and Crump, 2011; Fortunato et al., 2012; Portillo et al., 2012). More recently, however, there is growing interest in evaluating trait-based patterns of biogeography to better understand how and why particular community structures form and respond to environmental variation (Green et al., 2008; Boon et al., 2014).

Several important questions have arisen from characterizing microbial communities in the environment or in a host. These include: (1) “to what extent does a core microbial community exist?” and (2) “to what extent is variation in taxonomic community composition meaningful?” (Hamady et al., 2008). The size of taxonomic core microbial communities—those characterized based on the operational taxonomic units (OTUs) present—is dependent on sequence depth, where greater coverage results in larger numbers of OTUs associated with the core community in both environmental and host-associated samples (Qin et al., 2010; Caporaso et al., 2012b). However, identification of a core community offers little information as to the extent of interaction among community members, and functional redundancy among taxa suggests that taxonomically-defined communities may not allow for an adequate investigation of the microbial ecology of the system under study (Shade and Handelsman, 2012). Furthermore, the extent to which taxonomic variation influences functional diversity is not clear in the literature. For example, variation of taxonomic community structure among soil communities resulted in differences in the rate of litter decomposition and carbon dioxide production (Strickland et al., 2009), but trait convergence was observed among distinctly different communities associated with sponge species to select for specific functionality (e.g., denitrification) (Fan et al., 2012).

Functional redundancy among taxa and polyphyletic distribution of functional genes complicate the interpretation of core microbial communities and community variation when considering ecological processes and community stability (Boon et al., 2014). Several studies have failed to identify clear, host- or habitat-associated, taxonomic core communities (Burke et al., 2011; Huse et al., 2012), although 70% of functional similarity was observed in one study (Burke et al., 2011). Similarly, a study of cyanobacterial blooms among three distinct geographical regions revealed considerable phylogenetic variability among the bacterial communities, despite a high degree of consistency amongst functional traits observed (Steffen et al., 2012). Conversely, taxonomic core communities, often comprised of the most abundant OTUs, have been identified in several aquatic habitats (Gibbons et al., 2013; Staley et al., 2013). However, OTUs with low abundance, which have been found to comprise the majority of taxonomic diversity (Sogin et al., 2006), have been inferred to be the most active in a freshwater lake system (Jones and Lennon, 2010). Whether functionality among rare OTUs in aquatic systems is similar (i.e., minority members perform the same function) or variable (i.e., different minority taxa perform different functions) has been poorly explored thus far. A recent proposal suggests that intertwined ecological and evolutionary processes of selection, drift, dispersal, and mutation shape microbial biogeography (Hanson et al., 2012), and such a framework will allow for a better understanding of the role of core communities vs. less abundant taxa.

Microbial biogeography studies applying next-generation sequencing technologies targeting regions of the 16S rDNA have become relatively commonplace as the costs of next-generation sequencing rapidly decrease, but metagenomic studies necessary for characterization of functional diversity and identification of rare species still remain costly (Gilbert and Dupont, 2011; Knight et al., 2012). Recent estimates based on bacterial concentrations and average genome size suggest that metagenomic studies of seawater have sequenced <0.000001% of total DNA and that the sequencing of 4–5 × 10^9^ bp would provide coverage of only 0.001% in a 1L sample (Gilbert and Dupont, 2011). Similarly, the bacterial metagenome size in 1 g of soil has been estimated to comprise up to 3 × 10^15^ bp of DNA (Knight et al., 2012). Based on these estimates, the minimum sequencing effort to achieve 1× coverage of the bacterial metagenome of 1 L of seawater or 1 g of soil would require >800 and at least 5000 full runs, respectively, on a HiSeq 2000 platform, given the recently estimated sequence output (Caporaso et al., 2012a). Moreover, this sequencing effort would cost on the order of tens of millions of dollars.

Phylogenies of prokaryotes constructed from core genes, those present in nearly all sequenced members of a clade, are similar to those constructed from taxonomic marker genes (e.g., 16S rDNA) (Segata and Huttenhower, 2011). Processes including gene loss, convergent evolution, and lateral gene transfer complicate the relationship between phylogeny and function, but, generally, more complex traits (e.g., methanogenesis) are more likely to be isolated within only a few deep clades (Martiny et al., 2013). On the basis of the relationship between phylogeny and function, a computational approach (PICRUSt, phylogenetic investigation of communities by reconstruction of unobserved states) was devised to predict community functionality using 16S rDNA data and a reference database (Langille et al., 2013). PICRUSt metagenome predictions were strongly correlated (Spearman *r* = 0.82) with metagenome data from the Human Microbiome Project (HMP) and, while predictions were somewhat limited by sequencing depth, they were generally accurate even at a shallow depth of 16S rDNA sequencing (Langille et al., 2013). This subroutine was also shown to work well for more diverse soil samples which had a higher nearest sequence taxon index (NSTI {x} = 0.17 vs. 0.03 for HMP samples; *r* = 0.81, *P* < 0.001).

We previously characterized the taxonomic diversity of the Upper Mississippi River in Minnesota and found evidence of a core bacterial community comprised of highly abundant OTUs, with shifts in abundance potentially associated with variation in land cover (Staley et al., 2013). In the present study, we evaluated functional diversity throughout the Upper Mississippi River in Minnesota during the summer of 2012. Whole genome shotgun (metagenomic) sequencing was performed and these data were compared to functional inferences from 16S rRNA sequences obtained using PICRUSt. We anticipated that the majority of functional observations and predictions would be similar throughout the river based on the prevalence of the core bacterial community. We further hypothesized that fluctuations in the abundance of specific functional traits might be associated with variation in land cover types influencing water chemistry and the bacterial community. Since low sequence coverage was expected using the metagenomic shotgun approach, PICRUSt was used to infer functions among less abundant taxa that were likely to be absent from the metagenomic dataset. PICRUSt inferences were further compared against the shotgun metagenomic dataset to determine the accuracy of functional predictions. This study provides novel insight regarding the distribution of functional traits occurring within this riverine ecosystem and serves as one of the first studies to validate the use of PICRUSt in a diverse ecosystem.

## Materials and methods

### Sample collection, processing, and sequencing

**Figure 1:**
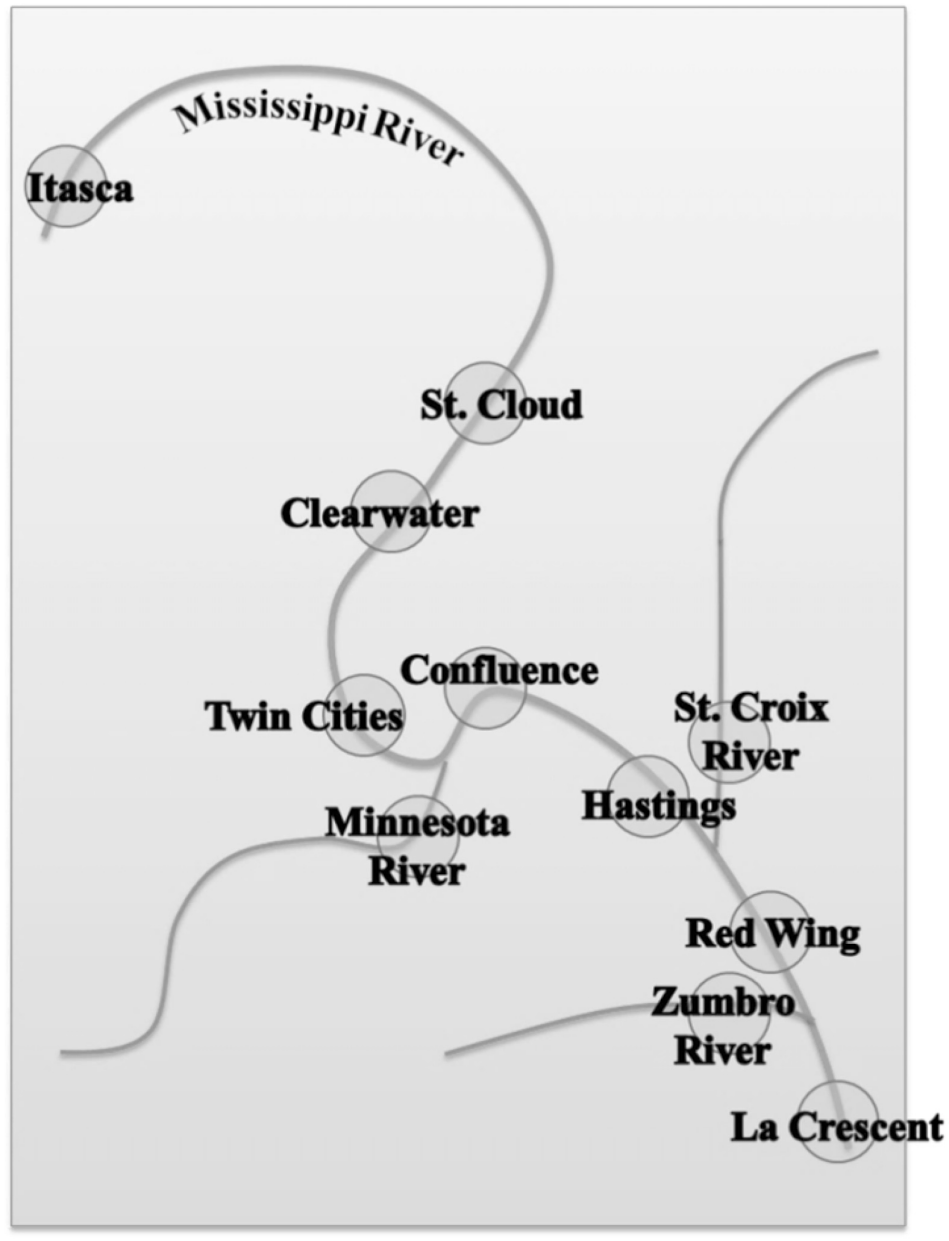
**Relative location of sampling sites along the Mississippi River and major contributing rivers**.

Samples were collected from May through July in 2012 from 11 sites along the Mississippi River and major contributing rivers beginning at the headwaters at Lake Itasca to the southern border of Minnesota, near La Crescent. The relative locations of sampling sites are shown in Figure 1, and exact sampling locations were previously described (Staley et al., 2013). Briefly, bacteria were concentrated from 40 L water samples on 0.45-μm-pore-size filters and elutriated by vortexing in pyrophosphate buffer, as described previously (Staley et al., 2013). We have previously compared the effect of filter pore size on the bacterial community characterized and found that this pore size allows for efficient filtration of large volumes of water used here (Staley et al., 2013), with minimal influence on overall estimates of community composition. A total of six cell pellets per site representing approximately 6–7 L of water each, were stored at −80°C until used. These pellets were used for a variety of experiments, not all of which are described here. DNA was extracted from cell pellets from each site (*n* = 11), 16S rDNA amplification, and sequencing were performed and reported in detail elsewhere (Staley et al., 2013), using the Metagenomic DNA Isolation Kit for Water (Epicentre, Madison, WI) and the 967F/1046R barcoded primer set (Sogin et al., 2006).

Whole genome shotgun sequences were generated using DNA extracted from separate cell pellets from each site (*n* = 11) using the MoBio PowerSoil® DNA Isolation Kit (Carlsbad, CA) according to the manufacturer's instructions. This kit was used to avoid inhibitors present in DNA prepared with the Epicentre kit and provided DNA at concentrations ranging from 10.7–44.7 ng μl^−1^. Where possible, samples were normalized to 20 ng μl^−1^ and 2 μg total DNA was submitted for sequencing. Sample quantities were normalized in libraries created at the University of Minnesota Genomics Center (UMGC) and libraries were paired-end sequenced at a read length of 100 bp and average insert size between 260 and 320 bp over two lanes on the HiSeq 2000 platform by the UMGC (St. Paul, MN). All sequences are deposited in the NCBI Sequence Read Archive under accession number SRP018728.

### Sequence processing and analysis

Metagenomic taxonomic data were exported directly from MG-RAST as summary tables of abundances of bacterial orders (Meyer et al., 2008). Taxonomic data from 16S sequences were derived using Mothur ver. 1.29 and our previously described processing pipeline (Schloss et al., 2009; Staley et al., 2013). Briefly, sequence reads were quality-trimmed using the following criteria: quality scores ≥35 in a 50 nt window, no ambiguous bases, and homopolymers ≤8 nt. Sequences containing primer mismatches were removed, as were singleton sequences. Chimeras were identified and removed using UCHIME (Edgar et al., 2011). Sequences were aligned against the SILVA database (Pruesse et al., 2007), OTUs were clustered at 97% similarity using the furthest-neighbor algorithm, and taxonomic assignment was performed using the Ribosomal Database Project ver. 9 database release (Cole et al., 2009).

Metagenomic sequence data was quality trimmed as described above (with the exception of primer matching and singleton removal) using Mothur software ver. 1.29 (Schloss et al., 2009). Quality-trimmed sequence data (mean 1.41 ± 0.36 Gb per sample) were uploaded to MG-RAST for taxonomic and functional annotations (Meyer et al., 2008), and these annotations are publically available in Project 3190. Due to higher sequence quality, only the forward read was used for analysis. Hierarchical functional predictions were performed using the KEGG Orthology (KO) database and default settings. The KO assignments were made in up to four tiers, where each tier is a more specific functional assignment. Data were exported as a QIIME report (Caporaso et al., 2010). To compare functional differences among sampling sites, abundances of predicted functions were normalized as percentages of the total number of predicted functions from the KO database (mean 2.89 ± 0.80 × 10^6^ reads per sample).

The 16S rDNA data were analyzed as indicated by the PICRUSt genome prediction software [http://picrust.github.io/picrust/] from raw sequence reads in the following environment: NumPy (1.7.1), biom-format (1.3.1), PyCogent (1.5.3), PICRUSt (1.0.0-dev), and PICRUSt script (1.0.0-dev). The number of reads per site was normalized by random subsample to 352,108 reads per sample, for both taxonomic and functional characterization. OTUs were assigned at 97% similarity, and 90.4% were mapped to the Greengenes ver. 13.5 database for functional prediction, with normalization to control for differences in 16S rDNA copy number among OTUs. Functional predictions were assigned up to KO tier 3 for all genes. To simplify analysis, however, only tier 1 functions of “metabolism,” “genetic information processing,” “environmental information processing,” and “cellular processes” were analyzed further, as the categories of “organismal systems” and “human disease” were thought to be poorly relevant to environmental samples. Results from the MG-RAST QIIME report were compared with predictions from PICRUSt using the “compare_biom.py” subroutine with normalization and observations not in the “expected data” file ignored. Results were compared using both shotgun metagenomic and PICRUSt predictions as the expected data.

### Statistical analyses

Spearman correlations relating abundances of shotgun metagenomic functional annotations, inferred functional abundances from PICRUSt, and taxonomic order abundances were performed using SPSS Statistics software ver. 19.0 (IBM, Armonk, NY). Taxonomic assignment was not performed to a more specific level (e.g., family or genus) because these assignments have been shown to be <90% accurate using short sequence reads (Mizrahi-Man et al., 2013). Comparisons of functional vs. taxonomic abundances were considered using data from the same source. Thus, shotgun metagenomic functional annotations were compared against taxonomic abundances derived from metagenome sequence data and functional inferences were compared against 16S rDNA taxonomic data. Differences in functional abundance between water basins containing more than one sampling site (*n* = 2–3) were compared via ANOVA, using the Statistical Analysis of Metagenomic Profiles (STAMP) software (Parks and Beiko, 2010). ANOVA analyses of differences in taxonomic order abundance were performed using SPSS software. All statistical analyses were evaluated at α = 0.05.

## Results

### Taxonomic characterization of bacterial communities

**Figure 2:**
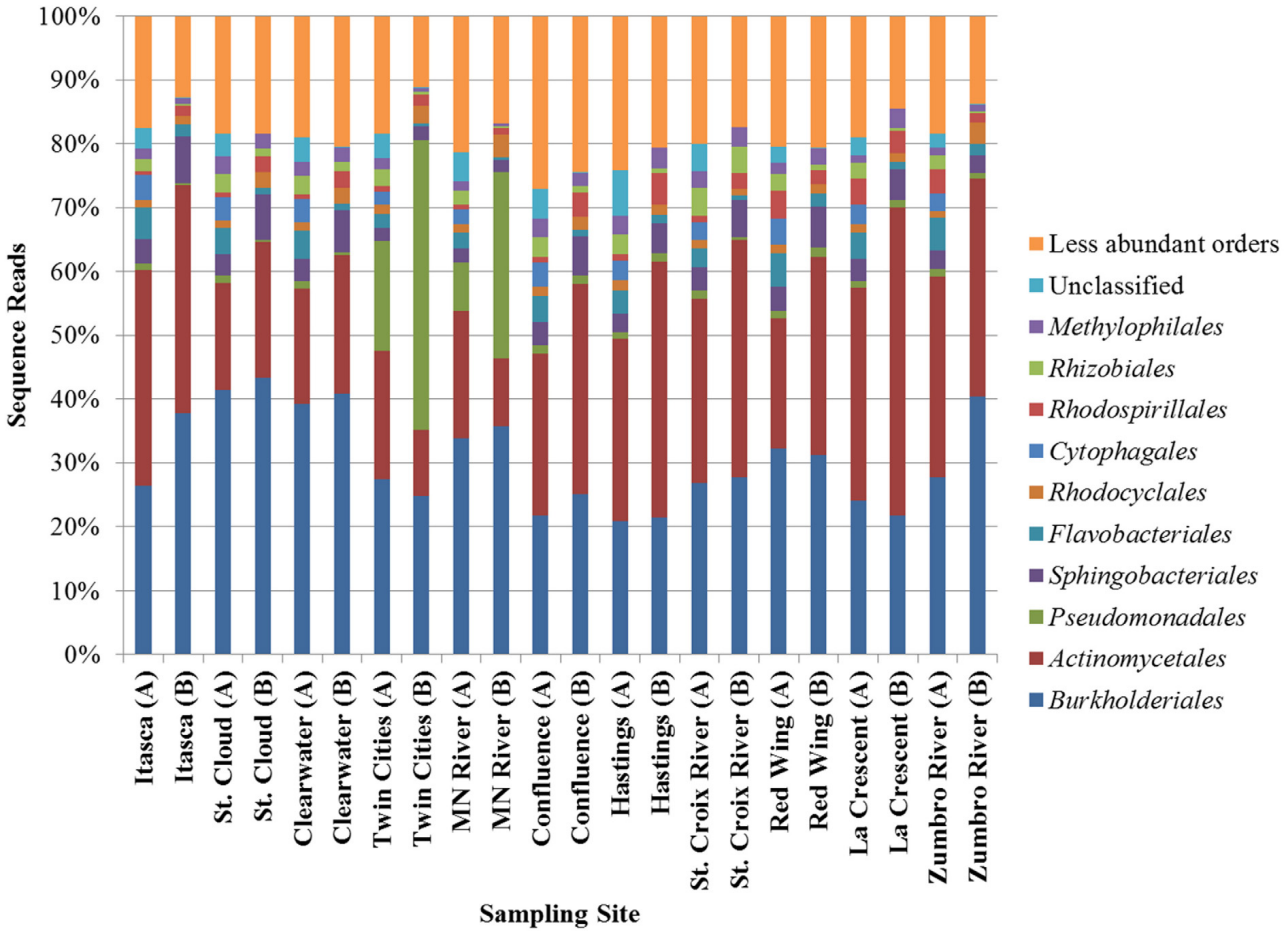
**Distribution of abundant orders determined by metagenomic shotgun sequencing (A) or 16S rRNA sequencing (B)**.

Bacterial communities characterized by both shotgun metagenomic analyses and 16S rDNA were similar and were dominated primarily by *Burkholderiales* and *Actinobacteria* (Figure 2). Relative abundances of orders of the α- and *γ-Proteobacteria* and the *Bacteroidetes* differed depending upon the sequencing method, but were also among the most numerous in all samples. The increased relative abundance of *Pseudomonadales* at the Twin Cities and Minnesota River sampling sites, as observed using 16S rDNA, was also observed, albeit to a lesser extent, in the metagenomic data. As expected, a higher percentage of sequence reads could not be classified to a specific order in the shotgun metagenomic dataset relative to the rDNA dataset. Non-bacterial orders, consisting primarily of orders belonging to the kingdoms *Plantae* and *Protista* and the phylum *Arthropoda*, were also identified in the metagenomic data, but these were present at very low abundances (<0.1% mean abundance among all samples). Bacterial community coverage using 16S rDNA was estimated at 99.0 ± 0.2% with a mean of 6752 ± 1589 OTUs at each site and a mean Shannon index of 4.33 ± 0.46. Notably, lower diversity was observed at the Twin Cities and Minnesota River sites (Shannon indices of 3.67 and 3.88).

### Functional characterization of bacterial communities

**Figure 3:**
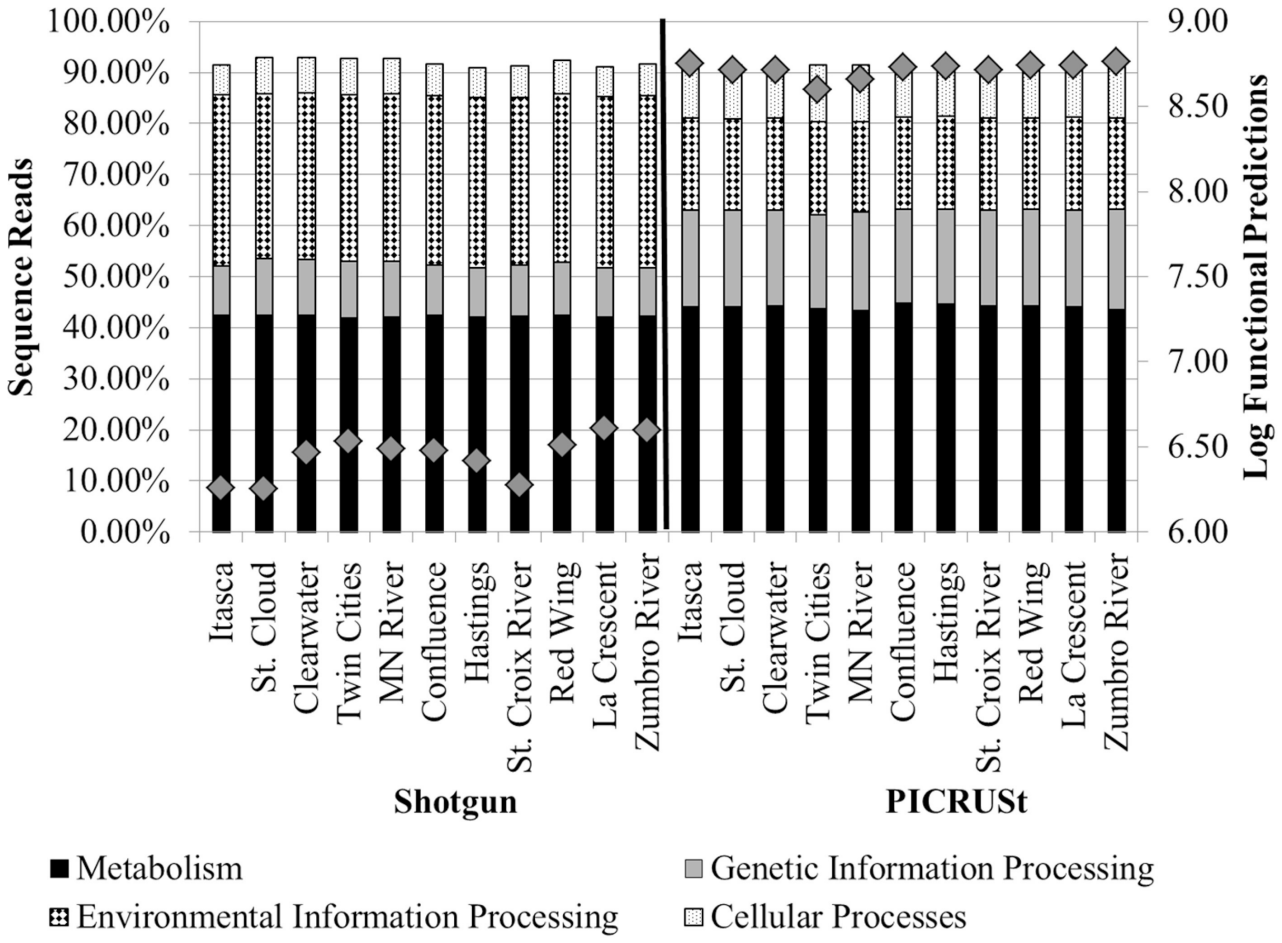
**Percentages of tier 1 KO functions among all shotgun metagenomic (left) and PICRUSt functional predictions (right)**. Functional categories for organismal systems and human diseases were omitted. Gray diamonds reflect the total number of annotated functional genes or PICRUSt predictions for all functional categories inferred at tier 2 (right y-axis).

The greatest number of genes (>40%) that were assigned a function encoded proteins involved in “metabolism” among tier 1 KO categories in both metagenomic and PICRUSt datasets (Table 1 and Figure 3). Annotation of the shotgun metagenomic dataset revealed >30% and ~10% of sequences encoded proteins involved in “environmental information processing” and “genetic information processing,” respectively (Figure 3). However, PICRUSt functional inferences revealed approximately the same percentages of genes in both of these functional categories, with 18.01 ± 0.16% and 18.88 ± 0.37% sequence reads per site for environmental and genetic information processing, respectively (Figure 3).

**Table 1:**
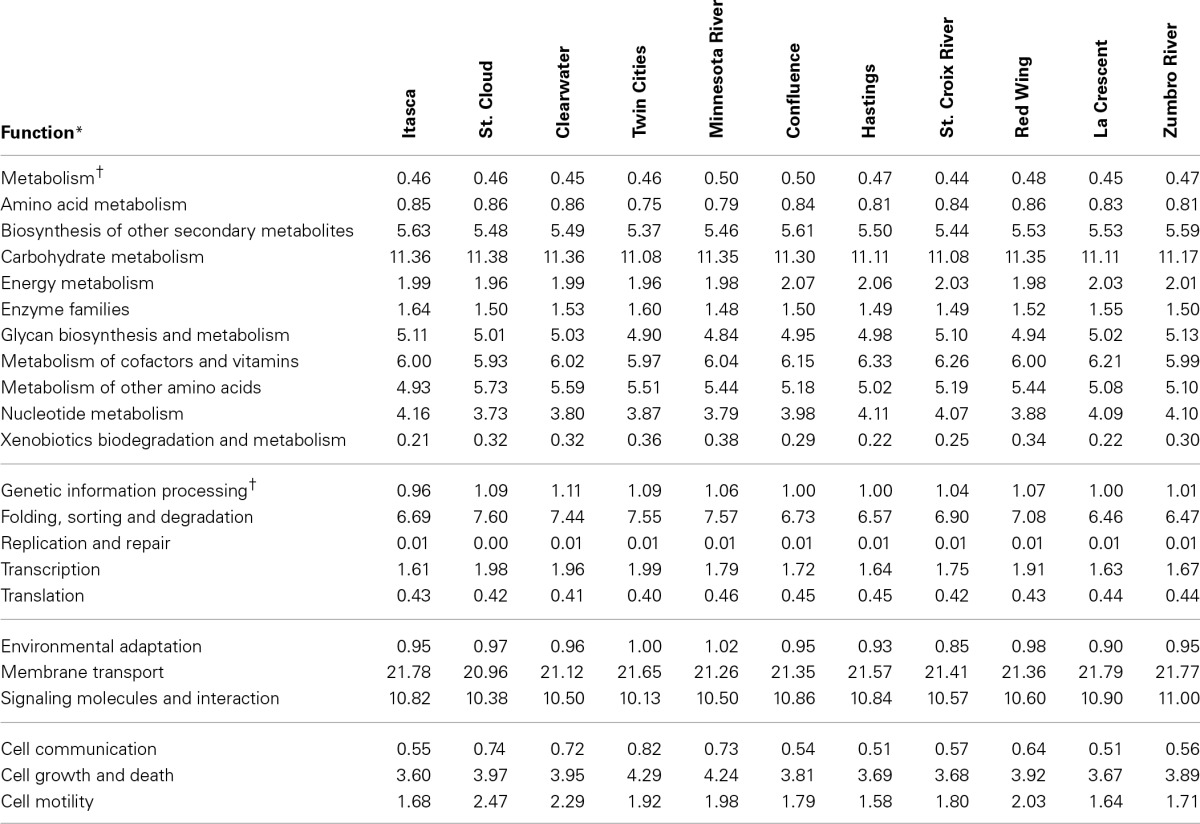
**Percentages of predicted sequences assigned to second tier KO categories in the metagenomic dataset**.

Thick lines separate tier 1 KO categories.

^*^Functional categories for which no reads were assigned are omitted.

^†^Predicted function only assigned at the first tier.

Notably, among sampling sites, shotgun metagenomic sequences showed little variation in the abundances and distributions of second- and third-tier KO functional gene annotations (Table 1). Among all sites, the highest standard deviation observed within a tier 2 functional category was 0.46% of sequence reads with a median of 0.02%. When third-tier functional categories were compared, the maximum standard deviation for a category, among all sites, was reduced to 0.42% with a median of 0.0002%. For PICRUSt predictions, median standard deviations were slightly higher—0.04 and 0.0057% for tier 2 and tier 3, respectively, with maxima of 0.46 and 0.70%.

### Taxonomic and land cover associations with functional traits

To determine which bacterial orders may be contributing to differences in functional traits among sites, correlation analyses were performed using both the shotgun metagenomic and PICRUSt datasets relating the 100 most abundant orders with second-tier functional classifications (Table S1). With the exception of translation, transcription, and membrane transport, a greater number of significant correlations (*P* < 0.05) were observed between functional and taxonomic abundances using PICRUSt functional predictions than were found using the shotgun metagenomic data, especially among low abundance orders. The majority of the PICRUSt correlations were positive, while the abundances of most functional classes in the metagenomic data (e.g., secondary metabolite biosynthesis and membrane transport) were negatively correlated with taxonomic abundance. Positive taxonomic-functional correlations among the PICRUSt data, however, are likely a result of autocorrelations as functional traits were predicted from taxonomic information. Although the abundances of many less abundant orders were positively correlated with PICRUSt-inferred functions, abundances of every second-tier function were also correlated with abundances of at least one of the most abundant orders identified in either dataset (Figure 2), most notably the *Actinomycetales* and *Flavobacteriales*.

Differences in functional community profiles from shotgun metagenomic data as well as those inferred from PICRUSt were further examined between water basins 070203 (St. Cloud and Clearwater sites; primarily agricultural land coverage) and 070206 (Twin Cities, Confluence, and Hastings; primarily developed or urbanized land coverage). Land cover for these basins was determined using the National Land Cover Database (Fry et al., 2011). These basins were the only ones containing multiple sites for statistical comparisons.

Among second-tier functional categories, the functional abundance of “amino acid metabolism” was significantly greater (*P* = 0.034) in the developed basin than the agricultural one (means of 21.52 and 21.04%, respectively), while the abundance of “glycan biosynthesis and metabolism” was greater in the agricultural basin (*P* = 0.024; developed mean of 1.76% vs. 2.38%), among shotgun metagenomic annotations. In contrast, by PICRUSt inference, the functional abundance of “glycan biosynthesis and metabolism” was significantly greater (*P* = 0.020) in the primarily agricultural basin vs. the developed one (means of 1.74 and 1.65% of genes inferred by PICRUSt, respectively). Significantly lower (*P* ≤ 0.035) functional abundance was observed for “amino acid metabolism,” “metabolism of terpenoids and polyketides,” and “protein folding, sorting and degradation” in the agricultural basin vs. the developed (means 10.97, 2.47, and 2.24% vs. 11.21, 2.60, 2.27%, respectively).

Abundances of bacterial orders that were correlated with functional traits which varied significantly in abundance between basins were further evaluated. Taxonomic-functional correlations observed among the shotgun metagenomic data (Table S1) were generally supported—greater abundance of a functional trait corresponded with significantly greater abundance of the taxa that were correlated with that trait. However, this was not observed among PICRUSt-inferred taxonomic-functional correlations (Table S1), and orders that were correlated with a functional trait were generally not more abundant in the basin favoring the trait. For example, *Actinomycetales*, *Bifidobacteriales*, and *Solirubrobacterales* were positively correlated with “amino acid metabolism” by shotgun metagenomic analyses, and all of these orders were significantly (*P* ≤ 0.043) more abundant in the developed basin. However, of the six orders inferred to be correlated with amino acid metabolism using PICRUSt, none of the orders were significantly different between basins based on 16S rDNA characterization.

### Comparison of metagenomic and PICRUSt functional prediction

Correlation analyses were performed to evaluate the relationship between percent abundances of second-tier functional gene categories in the shotgun metagenomic dataset and the percent abundances of genes inferred from PICRUSt. Site-specific differences in abundances of functional assignments were observed using PICRUSt, despite normalization to 352,108 sequence reads, with the log number of inferred functional assignments ranging from 8.06 to 8.76 per site (Figure 3). These differences in numbers of assignments are likely due to differences in copy number among the species identified at each site. Only six functional traits, “amino acid metabolism,” “energy metabolism,” “glycan biosynthesis and metabolism,” “transcription,” “signal transduction,” and “cell motility,” were significantly positively correlated between shotgun metagenomic and PICRUSt datasets (*r* = 0.696–0.825, *P* ≤ 0.032; Table 2). Notably, “enzyme families,” “genetic information processing,” “signaling molecules and interaction,” and “cellular processes and signaling” were identified by PICRUSt but not in the metagenomic dataset, and “cell communication” was the only functional trait identified in the shotgun metagenomic dataset but not by PICRUSt (Table 2). The percent abundances of all second-tier functional traits were significantly different between datasets via ANOVA (*P* ≤ 0.006). Among traits that differed by >5% between methods, “amino acid metabolism” and “translation” were higher in the shotgun metagenomic dataset, while PICRUSt inferred greater abundance of “membrane transport.”

**Table 2:** **Correlations of predicted second-tier functional abundances between metagenomic and PICRUSt annotations**.

**Function**		Metagenome (%)^*^	**PICRUSt (%)^†^**	**Spearman r**	***P*-value**
**1st tier**	**2nd tier**				
Metabolism	**Amino acid metabolism**	21.46 ± 0.28	11.15 ± 0.15	**0.825**	**0.032**
	Biosynthesis of other secondary metabolites	0.83 ± 0.03	0.96 ± 0.10	0.111	0.746
	Carbohydrate metabolism	11.24 ± 0.13	10.36 ± 0.34	−0.330	0.321
	**Energy metabolism**	6.08 ± 0.13	5.50 ± 0.15	**0.696**	**0.017**
	Enzyme families	ND	1.79 ± 0.04	NA	NA
	**Glycan biosynthesis and metabolism**	1.90 ± 0.28	1.63 ± 0.08	**0.797**	**0.003**
	Lipid metabolism	1.79 ± 0.15	4.28 ± 0.07	−0.023	0.947
	Metabolism of cofactors and vitamins	5.29 ± 0.26	4.12 ± 0.15	−0.513	0.107
	Metabolism of other amino acids	1.04 ± 0.05	2.07 ± 0.05	0.388	0.238
	Metabolism of terpenoids and polyketides	1.53 ± 0.05	2.57 ± 0.08	0.065	0.851
	Nucleotide metabolism	5.00 ± 0.09	3.01 ± 0.10	0.155	0.650
	Xenobiotics biodegradation and metabolism	0.63 ± 0.11	4.66 ± 0.23	0.425	0.193
Genetic Information Processing	Folding, sorting, and degradation	3.96 ± 0.15	2.23 ± 0.04	−0.220	0.516
	Genetic information processing	ND	2.06 ± 0.07	NA	NA
	Replication and repair	5.51 ± 0.08	6.76 ± 0.23	0.410	0.210
	**Transcription**	2.79 ± 0.09	2.32 ± 0.06	**0.741**	**0.009**
	Translation	10.64 ± 0.26	4.01 ± 0.11	0.256	0.447
Environmental Information Processing	Membrane transport	7.01 ± 0.46	12.40 ± 0.35	0.064	0.853
	Signaling molecules and interaction	ND	0.18 ± 0.01	NA	NA
	**Signal transduction**	3.88 ± 0.23	2.21 ± 0.16	**0.811**	**0.002**
Cellular Processes	Cell communication	0.01 ± 0.00	ND	NA	NA
	Cell growth and death	2.01 ± 0.04	0.44 ± 0.01	−0.081	0.813
	**Cell motility**	0.29 ± 0.06	2.49 ± 0.38	**0.804**	**0.003**
	Cellular processes and signaling	ND	3.27 ± 0.33	NA	NA
	Transport and catabolism	0.95 ± 0.05	0.37 ± 0.01	0.533	0.092

* Mean percent abundance of the functional trait among all samples by metagenomic analysis.

† Mean percent abundance of the functional trait among all samples by PICRUSt inference.

**Figure 4:**
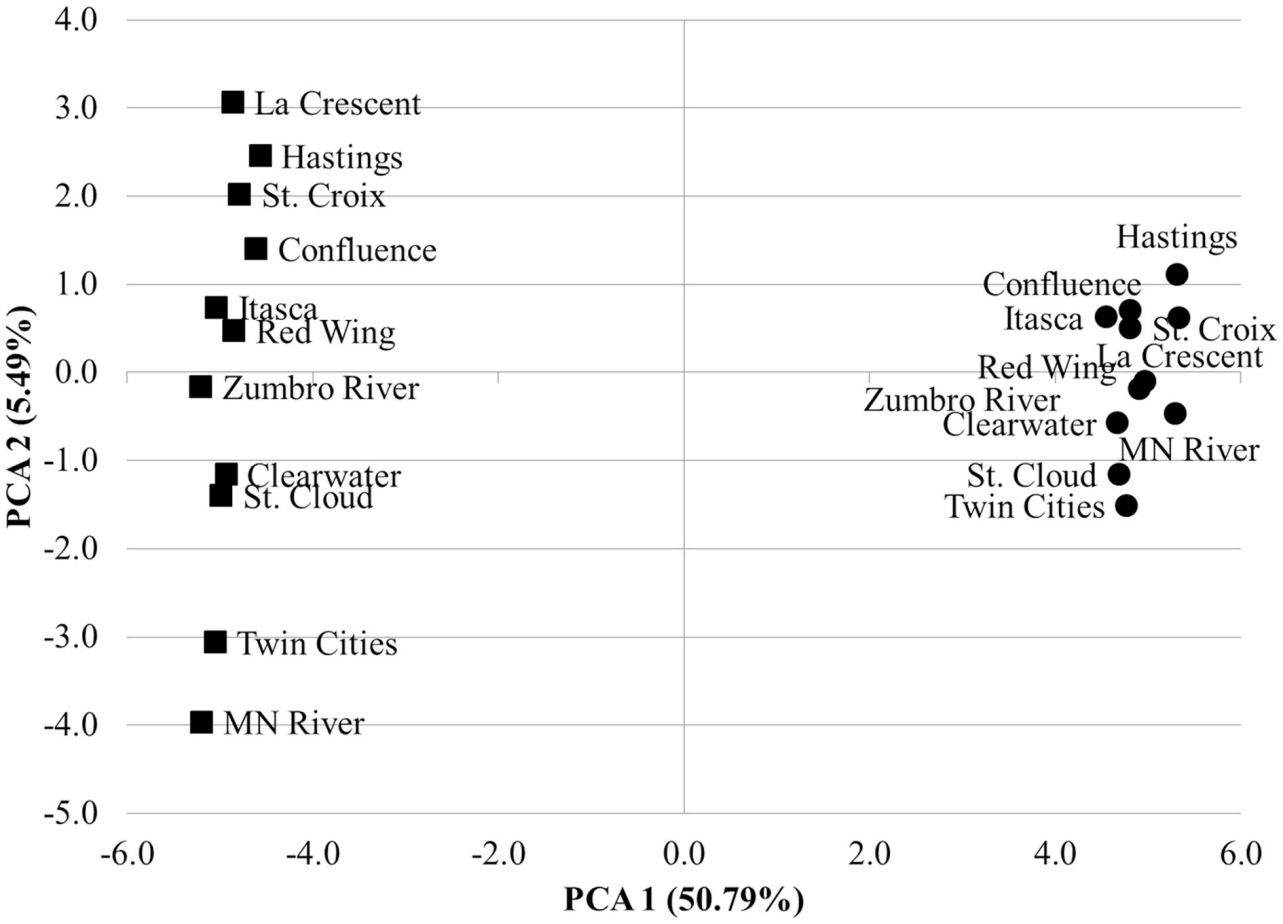
**Principal component analysis comparing 2nd tier KO functional traits derived from shotgun metagenomic analysis (circles) and PICRUSt-inference (squares)**. Traits were normalized as percentages of total genes assigned for both methods.

The mean nearest sequence taxon index (NSTI) of samples evaluated here was 0.11 ± 0.01, indicating PICRUSt inferences were likely to be better correlated with metagenomic data than the previously analyzed sediment dataset, that had a mean NSTI of 0.17 and correlation coefficient of 0.81 (Langille et al., 2013). Comparison of the accuracy of functional annotations among all sites using the “compare_biom.py” subroutine and the metagenomic dataset as the expected result revealed poor correlations between the two methods, and these correlations differed in direction among sampling sites (−0.017 ≤ r ≤ 0.022, mean *P* = 0.484 ± 0.227). PICRUSt had a mean sensitivity of 0.42 ± 0.01 and specificity of 0.58 ± 0.01 against the metagenomic results, suggesting only a moderate ability of PICRUSt to identify genes in the metagenomic dataset, while at the same time predicting many genes not found in metagenomic data. When metagenome data was validated against PICRUSt inferences, however, Spearman correlations were significant and positive for all sample sites (*r* = 0.135–0.165, *P* = 1.80 ± 5.31 × 10^−30^). The sensitivity of metagenomic annotations was 0.23 ± 0.02 and annotations were 0.88 ± 0.01 specific to PICRUSt inferences, indicating that most metagenomic functional annotations were included in the PICRUSt inferences and further revealing that only a small number of the PICRUSt inferences were accounted for by metagenomic analysis. Shotgun metagenomic data were further compared to PICRUSt-inferences via principal component analysis using all second-tier functional categories common to both datasets (Figure 4), and clear grouping of samples was observed based on the sequencing method used to determine the distribution of functional traits among samples.

## Discussion

We previously indicated that taxonomic characterization by sequencing of 16S rDNA suggests that the Mississippi River contains a stable, core bacterial community that was dominant throughout our study area in Minnesota (Staley et al., 2013). The metagenomic and PICRUSt-inferred data presented here further supports that the presence and distribution of functional traits are well conserved in this river system over a distance of >400 km. Due to the relatively low shotgun metagenomic coverage, however, it is likely that these “core functional genes” represent primarily those of the core bacterial community of highly abundant OTUs because functional genes of rare taxa were unlikely to be captured by shotgun sequencing. The high degree of matching (>90%) of 16S rDNA sequences to the GreenGenes database, though, suggests that at least some rare taxa were likely included in the functional inferences. Furthermore, a statistically significant correlation between PICRUSt inferences and shotgun metagenomic functional characterization, as well as nearly complete 16S rDNA coverage of samples, further supports that there were limited differences in the distribution of functional genes among sites. It should be noted that the ~10% of sequences that did not match the database may also possess unique functional genes important to highly-specialized processes occurring in this system.

Application of previous estimates of aquatic bacterial diversity (Gilbert and Dupont, 2011) to the sequence data generated here suggest that, at most, 0.00005% of the community DNA was annotated in this study. This estimate is likely very generous in relation to bacterial community coverage, specifically, as the DNA sequenced also included minor contributions from the *Archaea* and *Eukarya* that would further reduce coverage of the bacterial community alone. These contributions are nevertheless important as interactions among a large number of taxa at various trophic levels will impact specific bacterial responses (Verreydt et al., 2012), and several non-bacterial groups were significantly associated with the functional traits reported here.

Similar to shotgun metagenomic analysis, PICRUSt inferred relatively little functional variation among sampling sites. However, variation in specific functional traits inferred from PICRUSt was observed between two basins which varied greatly with respect to land coverage type, although only a few functional traits could be specifically associated with differences between the basins. A previous study of stream sediment bacterial communities found that the degree of urbanization, determined primarily as percentage of impervious cover, had significant impacts on community composition as well as functionality, measured as denitrification potential (Wang et al., 2011). Furthermore, this study found a link between the specific denitrifiers present and the community's ability to utilize various carbon substrates, suggesting functional variation may be due to both community membership and the influence of various land cover types. Similarly, in the present study, we hypothesize that functional variation between these basins may suggest that community functionality shifts in response to shifts in water chemistry associated with runoff from different land cover types. It was recently demonstrated, though, that land cover was related to water quality in an urban setting (Tu, 2011), so further study is necessary to better characterize land cover influences on microbial community function.

Interestingly, the least taxonomic bacterial diversity was observed at the Twin Cities and Minnesota River sites where the fewest functional predictions were determined via PICRUSt. It should be noted, however, that a decline in functionality at these sites should be interpreted cautiously as PICRUSt predictions were derived from taxonomic data (Langille et al., 2013). Lower taxonomic and, potentially, functional diversity, may be a result of biotic homogenization due to major pollutant discharges or introduction of non-indigenous bacteria in this more highly urbanized area, as was previously suggested for communities impacted by wastewater treatment effluent (Drury et al., 2013). However, another previous study also reported a correlation between 16S rDNA copy number and rapidity of response to resource availability among phylogenetically diverse taxa (Klappenbach et al., 2000), suggesting that high-copy-number (>5 copies) taxa are able to respond more rapidly to shifts in the environment. Furthermore, fewer functional predictions would be expected by PICRUSt for high-copy-number taxa due to normalization to copy number, although we cannot definitely conclude this is the reason for fewer functional predictions at these sites.

Interestingly, spikes in the relative abundance of the order *Pseudomonadales* were also observed at the Twin Cities and Minnesota River sites, and *Pseudomonas* spp. have been reported to have between 4 and 7 rRNA operons (Bodilis et al., 2012). It is possible that alteration in nutrient availability or introduction of highly competitive pseudomonads at these sites is the reason for the decreased taxonomic and functional diversity observed at these sites, although no significant differences in the abundance of discrete functional traits were observed at these sites by either sequencing method. Natural biological interactions may also explain the lower diversity observed, as eukaryotic activity was suggested to be associated with a *Vibrio* bloom in the English Channel that upset the usual seasonal community dynamics (Gilbert et al., 2012). Furthermore, similar *Pseudomonadales* spikes were observed at two upstream sites the previous year (data not shown), suggesting that this bloom may be a naturally-occurring phenomenon in this ecosystem in the early summer. The extent to which this bloom may impact the functional characteristics of the community remains to be studied, as the metagenomic sequencing depth here was too shallow to elucidate functions from less abundant taxa.

Despite the low shotgun metagenomic sequencing coverage, the abundances of minority orders were correlated with certain functional traits, most commonly those associated with transcription and translation. This finding suggests that these low abundance orders may be actively synthesizing proteins. Absence of a relationship between these orders and metabolic or environmental functions, though, may be due to low sequence coverage, as suggested above, or may indicate the presence of novel genes that are not yet represented in databases. The role of these low abundance taxa in community-level processes is likely to remain poorly understood for years to come, as the cost of next-generation sequencing remains prohibitively high to allow for much greater metagenomic coverage.

Tools such as PICRUSt, that are able to infer the distribution of functional traits among bacterial communities, offer an immediately-available method by which to evaluate community-level processes, but such tools should be well validated and interpretations of inferred results considered carefully. Results of the initial validation of PICRUSt suggested the tool was not effective in certain, high-diversity environments such as a hypersaline microbial mat that had a mean NSTI value of 0.23 and a poor correlation with metagenomic results (Spearman *r* = 0.25) (Langille et al., 2013). NSTI values for samples in this study indicated better suitability for PICRUSt analysis than sediment samples included in the initial validation, but correlation with metagenomic data was poorer than that observed for the hypersaline mat samples. Differences in sequence read length or sequencing target may explain the discrepancy between the previous study and the results presented here, as the majority of data used in the initial validation of PICRUSt targeted the V3–V5 region providing a longer read length (mean of 448 bp) compared to the V6 region sequenced here (<80 bp). In the current study, we found that PICRUSt showed imperfect sensitivity to metagenomic results. We suggest that this is a result of PICRUSt casting a wider net than did low-coverage metagenomic analysis—PICRUSt predicted potential functional traits of taxa from a 16S rDNA dataset and was more likely to capture minority members. However, due to their low abundance, the presence and potential functional traits of these rare taxa were less likely to be identified by the shallow metagenomic sequencing we performed.

It is difficult to determine if low correlations between datasets represent errors in PICRUSt inference or accurate functional predictions not detectable at shallow shotgun sequencing depth without more thorough metagenomic characterization. Due to the inherent uncertainty in inference-based functional annotation, tools such as PICRUSt may not be useful for high-resolution studies of functional biogeography in diverse ecosystems until their accuracy is better evaluated and/or databases are improved. Furthermore, differences in relative abundance between PICRUSt inferences and shotgun metagenomic data for every second-tier functional trait potentially indicate the PICRUSt inferences may not allow for accurate quantitative assessment of the distribution of functional traits in this ecosystem and those similar, although this should again be interpreted cautiously as greater metagenomic sequencing depth may have mitigated these differences.

Based on comparisons of taxonomic-functional correlations between annotation methods, the data presented here suggest the potential for a relatively high frequency of functional redundancy among members of this riverine bacterial community. A previous study characterizing functional and taxonomic diversity of carbon metabolism among freshwater habitats found that functional traits were primarily linked with nutrient concentrations, while taxonomic composition was more closely associated with habitat type (Comte and del Giorgio, 2009), similarly suggesting that functional redundancy exists among freshwater bacterial communities. Furthermore, among communities associated with the macroalga *Ulva australis*, 70% of functional similarity was maintained among several taxonomically diverse communities (Burke et al., 2011). Similarly, taxonomic community composition was shown to be variable in disparate freshwater cyanobacterial blooms, although the distribution of functional genes reminded highly consistent (Steffen et al., 2012). While the data gathered here do not allow assessment of differences in gene expression among community members, it seems reasonable that if an OTU performed a unique function, it would be found at increased abundance when the DNA associated with that function was more abundant. Considerably more bacterial orders were significantly and positively associated with function based on PICRUSt inference than were observed in the metagenomic data, and every functional trait inferred by PICRUSt was correlated with a high-abundance order. However, conclusions regarding taxonomic-functional correlations evaluated from PICRUSt inference should be interpreted cautiously as they potentially reflect an autocorrelation resulting from calculating taxonomic and functional abundances from a single dataset. As metagenomic sequencing was unlikely to reveal the presence of functional genes in low abundance orders, the potential for redundancy can only be hypothesized here and in light of previous studies (Comte and del Giorgio, 2009; Burke et al., 2011; Fan et al., 2012). Furthermore, the functional diversity within a single order can be very diverse, and better taxonomic resolution, resulting from longer sequence read lengths as next-generation sequencing technologies improve, may soon allow for more accurate family- or genus-level taxonomic assignment to better interrogate taxonomic-functional relationships.

Results of this study demonstrate that, at a relatively shallow sequencing depth, a core bacterial community maintains a high level of functional consistency in this riverine community. Although limited variation in the distribution of functional traits was observed among sampling sites and between water basins, high taxonomic diversity and functional redundancy may have limited our ability to detect and quantify differences in the distribution of functional traits among less abundant taxa. Nevertheless, slight, but significant, shifts were inferred in the distributions of community functional traits as a result of location, land coverage impacts, and/or other species sorting (environmental selection) dynamics, which appear to be influencing functional traits rather than specific taxa. Further investigation and validation of functional inference tools are necessary as metagenomic data continue to build in literature. As the costs of complete metagenomic community characterization remain prohibitively high, though, these tools may offer an important next step in evaluating major trends in trait-based microbial biogeography if databases can be expanded and taxonomic resolution improved.

### Conflict of interest statement

The authors declare that the research was conducted in the absence of any commercial or financial relationships that could be construed as a potential conflict of interest.
