# Supplementary material for: Core functional traits of bacterial communities in the Upper Mississippi River show limited variation in response to land cover
Source: Front Microbiol. 2014 Aug 8;5:414. doi: 10.3389/fmicb.2014.00414 (PMC4126211; doi:10.3389/fmicb.2014.00414)
Supplement: Supplementary file 1 [file DataSheet1.DOCX]

***Supplementary Material***

Core Functional Traits of Bacterial Communities in the Upper Mississippi River Show Limited Variation in Response to Land Cover

**Christopher Staley^1^, Trevor J. Gould^1,2^, Ping Wang^1^, Jane Phillips^2^, James B. Cotner^3^, and Michael J. Sadowsky^1,4,*^**

^1^BioTechnology Institute,

^2^Biology Program,

^3^Department of Ecology, Evolution and Behavior,

^4^Department of Soil, Water and Climate, University of Minnesota, St. Paul, MN

^*^Corresponding Author: Michael J. Sadowsky, BioTechnology Institute, University of Minnesota, 140 Gortner Lab, 1479 Gortner Ave, Saint Paul, MN 55108; Phone: (612)-624-2706, Email: [sadowsky@umn.edu](mailto:sadowsky@umn.edu)

**Supplementary Table**

Table S1. Spearman rank correlations relating taxonomic and functional abundances determined by metagenomic sequencing and functional traits inferred by PICRUSt. Thick lines divide first-tier KO functional categories.

| **Function** | **Correlated Orders (Metagenomic)^*^** | **Correlated Orders (PICRUSt)^*^** |
| --- | --- | --- |
| Amino acid metabolism | *Actinomycetales* (r = 0.645, *P* = 0.032)  *Bifidobacteriales* (r = 0.709, *P* = 0.015)  *Solirubrobacterales*  (r = 0.636, *P* = 0.035) | *Actinomycetales* (r = 0.773, *P* = 0.005)  *Flavobacteriales* (r = 0.855, *P* = 0.001)  *Neisseriales* (r = -0.809, *P* = 0.003)  *Chromatiales* (r = 0.727, *P* = 0.011)  *Halanaerobiales* (r = 0.782, *P* = 0.004)  *Lentisphaerales* (r = 0.626, *P* = 0.040) |
| Biosynthesis of other secondary metabolites | *Pseudomonadales* (r = -0.705, *P* = 0.015)  *Rhodocyclales* (r = -0.650, *P* = 0.030)  *Enterobacteriales* (r = -0.631, *P* = 0.037)  *Oceanospirillales* (r = -0.650, *P* = 0.030)  *Bifidobacteriales* (r -0.641, *P* = 0.034)  *Vibrionales* (r =-0.618, *P* = 0.043)  *Solirubrobacterales*  (r = -0.618, *P* = 0.043)  *Rhabditida*^An^ (r = -0.728, *P* = 0.011) | *Actinomycetales* (r =0.909, *P* < 0.001)  *Flavobacteriales* (r = 0.736, *P* = 0.010)  *Neisseriales* (r = -0.627, *P* = 0.039)  *Chromatiales* (r = 0.645, *P* = 0.032)  *Halanaerobiales* (r = 0.755, *P* = 0.007)  *Sphingomonadales*  (r = 0.664, *P* = 0.026)  *Enterobacteriales* (r = 0.655, *P* = 0.029)  *Lentisphaerales* (r = 0.607, *P* = 0.048)  *Ignavibacteriales* (r = 0.618, *P* = 0.043) |
| Carbohydrate metabolism | *Bifidobacteriales* (r = -0.615, *P* = 0.044)  *Acidimicrobiales* (r = -0.761, *P* = 0.006)  *Solirubrobacterales*  (r = -0.761, *P* = 0.006)  *Poales*^P^ (r = -0.839, *P* = 0.001) | *Actinomycetales* (r = 0.800, *P* = 0.003)  *Flavobacteriales* (r = 0.827, *P* = 0.002)  *Neisseriales* (r = -0.800, *P =* 0.003)  *Chromatiales* (r = 0.736, *P* = 0.010)  *Halanaerobiales* (r = 0.764, *P* = 0.006)  *Thiotrichales* (r = -0.627, *P* = 0.039) |
| Energy metabolism | *Poales*^P^ (r = 0.606, *P* = 0.048)  *Hymenoptera*^An^ (r = 0.638, *P* = 0.035)  *Haemospororida*^Pr^ (r = 0.629, *P* = 0.038) | *Actinomycetales* (r = 0.836, *P* = 0.001)  *Flavobacteriales* (r = 0.718, *P* = 0.013)  *Chromatiales* (r = 0.627, *P* = 0.039)  *Halanaerobiales* (r = 0.827, *P* = 0.002)  *Acidimicrobiales* (r = 0.645, *P* = 0.032)  *Enterobacteriales* (r = 0.618, *P* = 0.043)  *Pasteurellales* (r = 0.618, *P* = 0.043)  *Nitrosomonadales*  (r = 0.624, *P* = 0.040)  *Ignavibacteriales* (r = 0.773, *P* = 0.005)  *Acanthopleuribacterales*  (r = 0.638, *P* = 0.035) |
| Enzyme families |  | *Actinomycetales* (r = 0.682, *P* = 0.021)  *Flavobacteriales* (r = 0.891, *P* < 0.001)  *Neisseriales* (r = -0.782, *P* = 0.004)  *Chromatiales* (r = 0.636, *P* = 0.035)  *Halanaerobiales* (r = 0.645, *P* = 0.032)  *Thiotrichales* (r = -0.673, *P* = 0.023) |
| Glycan biosynthesis and metabolism | *Actinomycetales* (r = -0.700, *P* = 0.016)  *Bifidobacteriales* (r = -0.718, *P* = 0.013)  *Acidimicrobiales* (r = -0.645, *P* = 0.032) | *Sphingobacteriales*  (r = 0.627, *P* = 0.039)  *Rhodobacterales* (r = 0.609, *P* = 0.047)  *Gammaproteobacteria* *incertae sedis*  (r = 0.718, *P* = 0.013)  *Verrucomicrobiales*  (r = 0.773, *P* = 0.005)  *Oceanospirillales* (r = 0.655, *P* = 0.029)  *Verrucomicrobia* Subdivision 3  *genera incertae sedis*  (r = 0.664, *P* = 0.026)  *Bdellovibrionales* (r = 0.809, *P* = 0.003)  *Myxococcales* (r = 0.627, *P* = 0.039)  *Armatimonadales* (r = 0.673, *P* = 0.023)  *Bacteroidales* (r = 0.891, *P* < 0.001)  *Selenomonadales* (r = 0.864, *P* = 0.001)  *Thermales* (r = 0.945, *P* < 0.001)  *Nitrospirales* (r = 0.809, *P* = 0.003)  *Desulfuromonadales*  (r = 0.718, *P* = 0.013)  *Elusimicrobiales* (r = 0.764, *P* = 0.006)  *Deinococcales* (r = 0.618, *P* = 0.043)  *Ohtaekwangia* (r = 0.609, *P* = 0.047)  *Syntrophobacterales*  ( r = 0.855, *P* = 0.001)  *Holophagales* (r = 0.773, *P* = 0.005)  unclassified (r = 0.745, *P* = 0.008)  *Dehalogenimonas* (r = 0.655, *P* = 0.029)  *Desulfobacterales* (r = 0.800, *P* = 0.003)  *Syntrophorhabdaceae*  (r = 0.636, *P* = 0.035)  *Halobacteriales* (r = 0.682, *P* = 0.021)  *Thermoanaerobacterales*  (r = 0.764, *P* = 0.006)  *Methanobacteriales*  (r = 0.809, *P* = 0.003)  *Desulfovibrionales*  (r = 0.721, *P* = 0.012)  *Nautiliales* (r = 0.661, *P* = 0.027)  *Ignavibacteriales* (r = 0.609, *P* = 0.047)  *Aquificales* (r = 0.664, *P* = 0.026)  *Erysipelotrichales*  (r = 0.813, *P* = 0.002)  *Caldilineales* (r = 0.635, *P* = 0.036)  *Acidobacteria* Gp3  (r = 0.811, *P* = 0.002)  *Solirubrobacterales*  (r = 0.733, *P* = 0.010)  *Acidobacteria* Gp 22  (r = 0.711, *P* = 0.014)  *Acidobacteria* Gp 7(r = 0.655, *P* = 0.029)  *Coriobacteriales* (r = 0.752, *P* = 0.008)  *Desulfurellales* (r = 0.603, *P* = 0.050) |
| Lipid metabolism |  | *Actionmycetales* (r = 0.727, *P* = 0.011)  *Flavobacteriales* (r = 0.882, *P* < 0.001)  *Neisseriales* (r = -0.827, *P* = 0.002)  *Chromatiales* (r = 0.700, *P* = 0.016)  *Halanaerobiales* (r = 0.736, *P* = 0.010)  *Thiotrichales* (r = -0.627, *P* = 0.039) |
| Metabolism of cofactors and vitamins |  | *Actinomycetales* (r = 0.900, *P* < 0.001)  *Flavobacteriales* (r = 0.727, *P* = 0.011)  *Neisseriales* (r = -0.636, *P* = 0.035)  *Chromatiales* (r = 0.673, *P* = 0.023)  *Halanaerobiales* (r = 0.773, *P* = 0.055)  *Enterobacteriales* (r = 0.609, *P* = 0.047)  *Ignavibacteriales* (r = 0.664, *P* = 0.026) |
| Metabolism of other amino acids | *Burkholderiales* (r = 0.681, *P* = 0.021) | *Actinomycetales* (r = 0.627, *P* = 0.039)  *Flavobacteriales* (r = 0.909, *P* < 0.001)  *Neisseriales* (r = -0.764, *P* = 0.006)  *Chromatiales* (r = 0.618, *P* = 0.043)  *Halanaerobiales* (r = 0.736, *P* = 0.010)  *Syngergistales* (r = 0.673, *P* = 0.023)  *Enterobacteriales* (r = 0.645, *P* = 0.032)  *Lentisphaerales* (r = 0.653, *P* = 0.029) |
| Metabolism of terpenoids and polyketides |  | *Actinomycetales* (r = 0.827, *P* = 0.002)  *Flavobacteriales* (r = 0.800, *P* = 0.003)  *Neisseriales* (r = -0.845, *P* = 0.001)  *Chromatiales* (r = 0.800, *P* = 0.003)  *Halanaerobiales* (r = 0.718, *P* = 0.013)  *Thiotrichales* (r = -0.673, *P* = 0.023) |
| Nucleotide metabolism | *Chroococcales* (r = -0.764, *P* = 0.006)  *Nostocales* (r = -0.764, *P* = 0.006)  *Campylobacterales*  (r = -0.727, *P* = 0.011)  *Rickettsiales* (r = -0.618, *P* = 0.043)  *Oscillatoriales* (r = -0.736, *P* = 0.010)  *Gloeobacterales* (r = -0.609, *P* = 0.047)  *Rhabditida*^A^ (r = -0.609, *P* = 0.047) | *Actinomycetales* (r = 0.836, *P* = 0.001)  *Flavobacteriales* (r = 0.755, *P* = 0.007)  *Neisseriales* (r = -0.700, *P* = 0.016)  *Chromatiales* (r = 0.727, *P* = 0.011)  *Halanaerobiales* (r = 0.836, *P* = 0.001)  *Acidimicrobiales* (r = 0.627, *P* = 0.039)  *Ignavibacteriales* (r = 0.664, *P* = 0.026) |
| Xenobiotics biodegradation and metabolism | *Prochlorales* (r = -0.620, *P* = 0.042) | *Actinomycetales* (r = 0.745, *P* = 0.008)  *Flavobacteriales* (r = 0.809, *P* = 0.003)  *Neisseriales* (r = -0.882, *P* < 0.001)  *Chromatiales* (r = 0.791, *P* = 0.004)  *Halanaerobiales* (r = 0.664, *P* = 0.026)  *Alteromonadales* (r = -0.627, *P* = 0.039)  *Thiotrichales* (r = -0.727, *P* = 0.011)  *Cyanobacteria* Family XI  (r = -0.636, *P* = 0.035) |
| Folding, sorting, and degradation |  | *Actinomycetales* (r = 0.609, *P* = 0.047)  *Flavobacteriales* (r = 0.909, *P* < 0.001)  *Neisseriales* (r = -0.645, *P* = 0.032)  *Halanaerobiales* (r = 0.709, *P* = 0.015)  *Enterobacteriales* (r = 0.636, *P* = 0.035)  *Desulfobacterales* (r = 0.691, *P* = 0.019)  *Lentisphaerales* (r = 0.644, *P* = 0.033)  *Ignavibacteriales* (r = 0.782, *P* = 0.004) |
| Genetic information processing |  | *Flavobacteriales* (r = 0.809, *P* = 0.003)  *Halanaerobiales* (r = 0.682, *P* = 0.021)  *Selenomondales* (r = 0.691, *P* = 0.019)  *Thermales* (r = 0.736, *P* = 0.010)  *Desulfobacterales* (r = 0.655, *P* = 0.029) |
| Replication and repair | *Flavobacteriales* (r = 0.633, *P* = 0.036) | *Actinomycetales* (r = 0.818, *P* = 0.002)  *Flavobacteriales* (r = 0.773, *P* = 0.005)  *Neisseriales* (r = -0.736, *P* = 0.010)  *Chromatiales* (r = 0.736, *P* = 0.010)  *Halanaerobiales* (r = 0.845, *P* = 0.001)  *Lentisphaerales* (r = 0.603, *P* = 0.050) |
| Transcription | *Actinomycetales* (r = 0.779, *P* = 0.005)  unclassified (r = 0.683, *P* = 0.020)  *Clostridiales* (r = 0.679, *P* = 0.022)  *Myxococales* (r = 0.674, *P* = 0.023)  *Bifidobacteriales* (r = 0.743, *P* = 0.009)  *Lactobacillales* (r = 0.679, *P* = 0.022)  *Spirochaetales* (r = 0.688, *P* = 0.019)  *Desulfovibrionales*  (r = 0.665, *P* = 0.026)  *Thermoanaerobacterales*  (r = 0.743, *P* = 0.009)  *Thiotrichales* (r = 0.674, *P* = 0.023)  *Desulfobacterales* (r = 0.606, *P* = 0.048)  *Bacteroidetes* Order II *incertae sedis*  (r = 0.670, *P* = 0.024)  *Acidimicrobiales* (r = 0.793, *P* = 0.004)  *Thermales* (r = 0.679, *P* = 0.022)  *Deinococcales* (r = 0.697, *P* 0.017)  *Aquificales* (r = 0.647, *P* = 0.031)  *Prochlorales* (r = 0.779, *P* = 0.005)  *Thermotogales* (r = 0.643, *P* = 0.009)  *Fusobacteriales* (r = 0.665, *P* = 0.026)  *Selenomonadaes* (r = 0.697, *P* = 0.017)  *Saccharomycetales*^F^  (r = 0.752, *P* = 0.008)  *Rubrobacterales* (r = 0.702, *P* = 0.016)  *Coriobacteriales* (r = 0.743, *P* = 0.009)  *Solirubrobacterales*  (r = 0.683, *P* = 0.020)  *Methanosarcinales*  (r = 0.620, *P* = 0.042),  *Poales*^P^ (r = 0.624, *P* = 0.040)  *Diptera*^A^ (r = 0.747, *P* = 0.008)  *Dictyosteliida*^Pr^ (r = 0.724, *P* = 0.012)  *Chlamydomonadales*  (r = 0.642, *P* = 0.033)  *Brassicales*^P^ (r = 0.610, *P* = 0.046)  *Herpetosiphonales* (r = 0.656, *P* = 0.028)  *Synergistales* (r = 0.743, *P* = 0.009)  *Sphaerobacterales* (r = 0.743, *P* = 0.009)  *Erysipelotrichales* (r = 0.688, *P* = 0.019)  *Mycoplasmatales* (r = 0.733, *P* = 0.010)  *Halobacteriales* (r = 0.674, *P* = 0.023)  *Halanaerobiales* (r = 0.670, *P* = 0.024)  *Haemosporida*^Pr^ (0.674, *P* = 0.023)  *Ktedonobacterales* (r = 0.670, *P* = 0.024) | *Actinomycetales* (r = 0.800, *P* = 0.003)  *Flavobacteriales* (r = 0.827, *P* = 0.002)  *Neisseriales* (r = -0.800, *P* = 0.003)  *Chromatiales* (r = 0.736, *P* = 0.010)  *Halanaerobiales* (r = 0.764, *P* = 0.006)  *Thiotrichales* (r = -0.627, *P* = 0.039) |
| Translation | *Actinomycetales* (r = 0.743, *P* = 0.009)  *Flavobacteriales* (r = 0.661, *P* = 0.027)  *Sphingobacteriales*  (r = 0.656, *P* = 0.028)  *Clostridiales* (r = 0.715, *P* = 0.013)  *Bacillales* (r = 0.706, *P* = 0.015)  *Myxococcales* (r = 0.683, *P* = 0.020)  *Bifidobacteriales* (r = 0.661, *P* = 0.027)  *Lactobacillales* (r = 0.715, *P* = 0.013)  *Spirochaetales* (r = 0.738, *P* = 0.010)  *Desulfovibrionales* (r = 0.661, *P* = 0.027)  *Thermoanaerobacterales*  (r = 0.724, *P* = 0.012)  *Chloroflexales* (r = 0.606, *P* = 0.048)  *Bacteroidetes* Order II *incertae sedis*  (r = 0.724, *P* = 0.012)  *Acidimicrobiales* (r = 0.661, *P* = 0.027)  *Aquificales* (r = 0.670, *P* = 0.024)  *Prochlorales* (r = 0.679, *P* = 0.022)  *Thermotogales* (r = 0.724, *P* = 0.012)  *Hydroida* (r = 0.642, *P* = 0.033)  *Fusobacteriales* (r = 0.661, *P* =0.027)  *Selenomonadales* (r = 0.706, *P* = 0.015)  *Saccharomycetales*^F^  (r = 0.706, *P* = 0.015)  *Rubrobacterales* (r = 0.720, *P* = 0.013)  *Coriobacteriales* (r = 0.724, *P* = 0.012)  *Methanosarcinales*  (r = 0.661, *P* = 0.027)  *Diptera*^A^ (r = 0.692, *P* = 0.018)  *Dictyosteliida*^Pr^ (r = 0.733, *P* = 0.010)  *Puniceicoccales* (r = 0.697, *P* = 0.017)  *Brassicales*^P^ (r = 0.665, *P* = 0.026)  *Herpetosiphonales* (r = 0.692, *P* = 0.018)  *Synergistales* (r = 0.724, *P* = 0.012)  *Sphaerobacterales* (r = 0.724, *P* = 0.012)  *Erysipelotrichales* (r = 0.738, *P* = 0.010)  *Mycoplasmatales* (r = 0.756, *P* = 0.007)  *Halobacteriales* (r = 0.706, *P* = 0.015)  *Halanaerobiales* (r = 0.706, *P* = 0.015)  *Eurotiales*^F^ (r = 0.606, *P* = 0.048)  *Ktedonobacterales* (r = 0.688, *P* = 0.019) | *Actinomycetales* (r = 0.755, *P* = 0.007)  *Flavobacteriales* (r = 0.782, *P* = 0.004)  *Halanaerobiales* (r = 0.818, *P* = 0.002)  *Enterobacteriales* (r = 0.682, *P* = 0.021)  *Desulfuromonadales*  (r = 0.618, *P* = 0.043)  *Nitrosomonadales*  (r = 0.624, *P* = 0.040)  *Desulfobacterales* (r = 0.636, *P* = 0.035)  *Lentisphaerales* (r = 0.708, *P* = 0.015)  *Ignavibacteriales* (r = 0.800, *P* = 0.003)  *Acanthopleuribacterales*  (r = 0.606, *P* = 0.048) |
| Membrane transport | *Actinomycetales* (r = -0.727, *P* = 0.011)  *Clostridiales* (r = -0.627, *P* = 0.039)  *Bacillales* (r = -0.636, *P* = 0.035)  *Bifidobacteriales* (r = -0.709, *P* = 0.015)  *Lactobacillales* (r = -0.627, *P* = 0.039)  *Spirochaetales* (r = -0.618, *P* = 0.043)  *Thermoanaerobacterales*  (r = -0.673, *P* = 0.023)  *Acidimicrobiales* (r = -0.691, *P* = 0.019)  *Thermales* (r = -0.627, *P* = 0.039)  *Deinococcales* (r = -0.636, *P* = 0.035)  *Prochlorales* (r = -0.636, *P* = 0.035)  *Thermotogales* (r = -0.673, *P* = 0.023)  *Selemonadales* (r = -0.636, *P* = 0.035)  *Saccaharomycetales*^F^  (r = -0.645, *P* = 0.032)  *Rubrobacterales* (r = -0.691, *P* = 0.019)  *Coriobacterales* (r = -0.673, *P* = 0.023)  *Solirubrobacterales*  (r = -0.636, *P* = 0.035)  *Dictyosteliida*^Pr^ (r = -0.664, *P* = 0.026)  *Puniceicoccales* (r = -0.618, *P* = 0.043)  *Brassicales*^P^ (r = -0.618, *P* = 0.043)  *Synergistales* (r = -0.673, *P* = 0.023)  *Sphaerobacterales*  (r = -0.673, *P* = 0.023)  *Eryspielotrichales*  (r = -0.618, *P* = 0.043)  *Mycoplasmatales* (r = -0.655, *P* = 0.029)  *Halobacteriales* (r = -0.655, *P* = 0.029)  *Ktedonobacterales*  (r = -0.618, *P* = 0.043) | *Flavobacteriales* (r = 0.945, *P* < 0.001)  *Neisseriales* (r = -0.755, *P* = 0.007)  *Halanaerobiales* (r = 0.700, *P* = 0.016)  *Syngeristales* (r = 0.664, *P* = 0.026)  *Enterobacteriales* (r = 0.655, *P* = 0.029)  *Lentisphaerales* (r = 0.621, *P* = 0.041)  *Ignavibacteriales* (r = 0.609, *P* = 0.047) |
| Signal transduction | *Burkholderiales* (r = 0.664, *P* = 0.026) | *Burkholderiales* (r = 0.727, *P* = 0.011)  *Flavobacteriales* (r = 0.609, *P* = 0.047)  *Thermales* (r = 0.709, *P* = 0.015)  *Methanobacteriales*  (r = 0.664, *P* = 0.026) |
| Cell communication |  | *Rhizobiales* (r = 0.798, *P* = 0.003)  *Hydrogenophilales*  (r = 0.615, *P* = 0.044)  *Verrucomicrobia* Subdivision 3  *genera incertae sedis*  (r = 0.651, *P* = 0.030)  *Opitutales* (r = 0.679, *P* = 0.022)  *Methylococcales* (r = 0.725, *P* = 0.012)  *Sneathiellales* (r = 0.607, *P* = 0.048)  *Acidobacteria* Gp 6  (r = 0.789, *P* =0.004)  *Gemmatimonadales*  (r = 0.615, *P* = 0.044)  *Myxococcales* (r = 0.688, *P* = 0.019)  *Legionellales* (r = 0.734, *P* = 0.010)  *Bryobacter* (r = 0.826, *P* = 0.002)  *Nitrospirales* (r = 0.771, *P* = 0.006)  *Syntrophobacterales*  (r = 0.734, *P* = 0.010)  *Anaerolinales* (r = 0.881, *P* < 0.001)  *Lactobacillales* (r = 0.878, *P* < 0.001)  *Halobacteriales* (r = 0.679, *P* = 0.022)  *Thermoanaerobacterales*  (r = 0.688, *P* = 0.019)  *Acidobacteria* Gp 2  (r = 0.759, *P* = 0.007)  *Erysipelotrichales*  (r = 0.641, *P* = 0.034)  *Deferribacterales* (r = 0.657, *P* = 0.028)  *Edaphobacter* (r = 0.945, *P* < 0.001)  *Alphaproteobacteria incertae sedis*  (r = 0.644, *P* = 0.033) |
| Cell growth and death | *Acidimicrobiales* (r = 0.665, *P* = 0.026) | *Flavobacteriales* (r = 0.800, *P* = 0.003)  *Halanaerobiales* (r = 0.845, *P* = 0.001)  *Enterobacteriales* (r = 0.709, *P* = 0.015)  *Thermales* (r = 0.609, *P* = 0.047)  *Desulfuromonadales*  (r = 0.682, *P* = 0.021)  *Desulfobacterales* (r = 0.745, *P* = 0.008)  *Erysipelotrichales*  (r = 0.644, *P* = 0.033)  *Desulfurellales* (r = 0.703, *P* = 0.016) |
| Cell motility | *Burkholderiales* (r = 0.753, *P* = 0.007)  *Rhodocyclales* (r = 0.635, *P* = 0.036)  *Campylobacteriales*  (r = 0.648, *P* = 0.031) | *Burkholderiales* (r = 0.927, *P* < 0.001)  *Cyanobacteria* (unclassified)  (r = -0.664, *P* = 0.026)  *Methanobacteriales*  (r = 0.645, *P* = 0.032) |
| Cellular processes and signaling |  | *Burkholderiales* (r = 0.773, *P* = 0.005)  *Thermales* (r = 0.718, *P* = 0.013)  *Methanobacteriales*  (r = 0.673, *P* = 0.023) |
| Transport and catabolism | *Burkholderiales* (r = 0.624, *P* = 0.040)  *Campylobacterales*  (r = 0.624, *P* = 0.040) | *Flavobacteriales* (r = 0.945, *P* < 0.001)  *Neisseriales* (r = -0.755, *P* = 0.007)  *Halanaerobiales* (r = 0.700, *P* = 0.016)  *Syngeristales* (r = 0.664, *P* = 0.026)  *Enterobacteriales* (r = 0.655, *P* = 0.029)  *Lentisphaerales* (r = 0.621, *P* = 0.041)  *Ignavibacteriales* (r = 0.609, *P* = 0.047) |

^*^Orders are listed in order from most to least abundant.

^A^Order belongs to Kingdom *Animalia.*

^F^Order belongs to Kingdom *Fungi.*

^P^Order belongs to Kingdom *Plantae.*

^Pr^Order belongs to Kingdom *Protista.*
